# The association between family impact and health-related quality of life of children with idiopathic central precocious puberty in Chongqing, China

**DOI:** 10.1186/s12955-021-01805-w

**Published:** 2021-06-26

**Authors:** Hong Yang, Shunqing Luo, Xiaohua Liang, Qin Lin, Ting Cheng, Li Zeng, Fang Tang, Di Wu

**Affiliations:** 1 Department of General Medicine, National Clinical Research Center for Child Health and Disorders, Ministry of Education Key Laboratory of Child Development and Disorders, Chongqing Key Laboratory of Pediatrics, Children’s Hospital of Chongqing Medical University, Jinyu Avenue No. 20, Yubei, Chongqing, 400010 China; 2 Clinical Epidemiology and Biostatistics Department, National Clinical Research Center for Child Health and Disorders, Ministry of Education Key Laboratory of Child Development and Disorders, Chongqing Key Laboratory of Pediatrics, China International Science and Technology Cooperation Center of Child Development and Critical Disorders, Children’s Hospital of Chongqing Medical University, Zhongshan 2nd Road No. 136, Yuzhong, Chongqing, 400016 China; 3 Department of Endocrinology and Internal Medicine, National Clinical Research Center for Child Health and Disorders, Ministry of Education Key Laboratory of Child Development and Disorders, Chongqing Key Laboratory of Pediatrics, Children’s Hospital of Chongqing Medical University, Zhongshan 2nd Road No. 136, Yuzhong, Chongqing, 400016 China

**Keywords:** Precocious puberty, Child, Quality of life, Family impact

## Abstract

**Background:**

Idiopathic central precocious puberty (ICPP) reduces patient health-related quality of life (HRQoL). The impacts of disease and treatment on families are also an important concern. This study aimed to assess the association between family impact and HRQoL of children with ICPP.

**Methods:**

We conducted a case–control study in Chongqing, China. A case group of 134 children with ICPP aged 5–12 years and their caregivers was recruited from a children’s hospital in Chongqing. A total of 210 gender- and age-matched subjects from two primary schools were selected as controls. PedsQL^TM^4.0 Generic Core Scales (GCS) and PedsQL™ Family Impact Module (FIM) were used in this study.

**Results:**

Children with ICPP scored lower than controls in all HRQoL domains except physical functioning. In particular, the two groups were significantly different in emotional functioning scores (*d* = 0.414, *P* < 0.001). Compared with controls, ICPP families had lower scores in all dimensions of the FIM scale (*d* = 0.288–1.030, all *P* < 0.05). Factors associated with HRQoL of ICPP patients included: age of patients, type of medical treatment, employment status of caregivers, educational level of caregivers, parent HRQoL and family functioning (all *P* < 0.05).

**Conclusions:**

Children with ICPP demonstrated lower quality of life and greater family impact compared to healthy controls. In addition, less impact of disease on parent HRQoL and family functioning was associated with better HRQoL of ICPP patients, patients aged older, treated with drug combination, cared by employed or well-educated caregivers reported better HRQoL. Health care professionals should pay more attention to younger patients treated with GnRHa alone, and provide targeted interventions for caregivers depending on their demographic background to reduce family impact and thereby improve patient HRQoL.

## Background

Idiopathic central precocious puberty (ICPP) is defined as the development of secondary sexual characteristics following the activation of the hypothalamic–pituitary–gonadal axis before age 8 in girls and age 9 in boys of unknown etiology after a diagnostic evaluation [1], with an annual incidence of 1/5000 to 1/10,000 investigated by National Institute of Child Health and Human Development in 1982 [2]. The incidence of precocious puberty is increasing in recent years with the rapid economic development. A cross-sectional survey in Denmark showed that 0.2% of girls and < 0.05% boys had experienced some form of precocious puberty compared with 4.5 million people in 1961 [3]. A cross-sectional epidemiological study in China found that the prevalence of precocious puberty was 0.43%, and it was higher in northern region than that in southwest region [4].

Gonadotropin releasing hormone agonists (GnRHa) have become gold-standard treatments for ICPP since the mid-1980s, with main therapeutic goal of improving final height [5]. Numerous studies showed that GnRHa therapy can obviously improve adult final height [6, 7], but the consensus on the use of GnRHa also stressed the importance of preventing mental and social problems associated with sexual development [5]. ICPP patients had a more obese body image and an exaggerated sense of breast development compared to their peers [8], which made them demonstrate significantly higher emotional reactivity in social life [9], they tended to be shy, lonely, and often feel inferior, moody, or sad [10]. Some patients even demonstrated body image dissatisfaction after 12 months of GnRHa treatment [11]. Therefore, under the background of bio-psycho-social model, it is necessary for clinic staff to comprehensive evaluate patients’ health status that go beyond physical well-being.

Parents of children with ICPP are confronted by the challenges of chronic disease management, such as prolonged illness, frequent re-examinations, and cost burdens, they were likely to express anxiety and guilt when caring for children with ICPP [12]. A study using the World Health Organization quality of life questionnaire found that parents of children with ICPP had lower scores on physical and mental health domains than controls [13]. An American study reached similar conclusions; caregivers reported anxiety and guilt while explaining the diagnosis and treatment process to their child, and also expressed a desire to affiliate with others experiencing similar struggles [14].

Health-related quality of life (HRQoL) accords with modern medical model, it multiple assesses individual health status including physical, emotional and social health. But only a few studies focused on the impact of ICPP on the HRQoL of children, and found that ICPP patients had lower quality of life during treatment [15, 16]. Parents caring a children with ICPP expressed negative emotions, and family bore economic pressure, but the impact ICPP on caregivers’ HRQoL and family functioning has not been studied in China. Consequently, the aim of this study is to assess the impact of ICPP on the HRQoL of children and their parents and on family functioning in Chongqing, China, and to determine the association between family impact and HRQoL in ICPP patients. Study findings could potentially provide a perspective to inform the development of clinical interventions to improve the HRQoL of children with ICPP.

## Methods

### Participants and setting

To achieve the power (1 − *β*) of 0.80 under the probability of type I error (*α*) of 0.05, hypothetical mean scores of Psychosocial Health Summary Score were 82.0 and 87.0 in the case and control groups, respectively, with a standard deviation of 15. The sample size was determined by using the formula of *n* = (*σ*((*z*_1 *− α/*2_ + *z*_1 *− β*_)/(*μ*_*A*_ − *μ*_*B*_)))^2^ [17]. A minimum of 139 subjects was required for the case and control groups, respectively. A cohort of 134 children with ICPP and their primary caregivers was recruited from the Children’s Hospital of Chongqing Medical University. The hospital is one of National Regional Medical Center for Children in China, which has enough ICPP cases. According to the previous results that age of diagnosis of ICPP mainly focused on 7–10 years [18, 19], finally, in the 12 selected classes of two elementary schools, 230 gender- and age-matched children and their caregivers were selected in Chongqing. Of these, 215 agreed to participate and 15 refused for varying reasons. 210 children and their caregivers had completed all the questionnaires and included in the final study.

The case group met the following inclusion criteria [20]: (1) new diagnosis of ICPP or previously diagnosed ICPP undergoing treatment, diagnostic criteria of ICPP were as follows: ① Appearance of secondary sexual characteristics before 8 years in girls or 9 years in boys, and patients whose progression of sexual development from one pubertal stage to the next in 3–6 months were also included although their secondary sexual characteristics began to develop after the age of 8 years in girls or 9 years in boys; ② height velocity above the 97th percentile for the age; ③ Bone age is generally advanced by 1 year or more in relation to chronological age; ④ Uterine length ≥ 3.4 cm, ovarian volume ≥ 1 mL, and at the same time, at least one ovarian follicle’s diameter > 4 mm in girls, testicular volume ≥ 4 ml or length of > 2.5 cm in boys; ⑤ Concentrations of LH peak is higher than 5 IU/L and of LH-to-FSH ratio is higher than 0.6 in the GnRH stimulation test. (2) patients aged between 5 and 12 years. Exclusion criteria were: (1) chronic illnesses such as asthma, kidney or heart diseases, epilepsy, or other comorbidities that influence HRQoL; (2) a history of psychological trauma such as the death of a family member; or (3) refusal of either the patient or their primary caregivers to participate.

The control group met the following inclusion criteria: (1) children aged between 5 and 12 years; (2) secondary sexual characteristic appearance was not observed by inspection and palpation according to the Marshall and Tanner method, and (3) voluntary participation of the patient and their caregivers. We excluded children with any of the following conditions: (1) children with recent or past diagnoses of ICPP; (2) chronic illnesses such as asthma, kidney or heart diseases, epilepsy, or other comorbidities that influence HRQoL; or (3) a history of psychological trauma such as the death of a family member. This study was approved by the Ethics Committee of Children’s Hospital of Chongqing Medical University. All caregivers provided written informed consent.

### Procedure

The investigators were trained before data collection to ensure the quality of clinical research practices. In the case group, parents completed a demographic questionnaire and the PedsQL™ FIM, whereas patients completed the PedsQL^TM^4.0 GCS at the time of diagnosis or during outpatient follow-up. In the control group, children completed the PedsQL^TM^4.0 GCS, and brought the demographic questionnaire and the PedsQL^TM^4.0 FIM to their parents for completion, which were returned to the head teacher on the following day, and were then collected by the investigators.

### Measures

Demographic variables included age, gender, weight, height; number of children in the family; and caregiver relationship (e.g., parents or grandparents). The caregivers’ basic information included living environment (e.g., urban, rural), educational level (primary school, middle school, high school, or university and higher), employment status (employed, unemployed/housewives) and monthly income (< 5000, 5,000–10,000, > 10,000 Chinese Yuan [CNY]). Clinical characteristics of children with ICPP included whether they were newly diagnosed or already undergoing follow-up; age at diagnosis; disease duration; type of treatment (e.g., GnRHa or GnRHa combined with growth hormone); route of administration (e.g., intramuscular or subcutaneous injection); and duration of treatment.

The PedsQL™ GCS questionnaire was developed by Varin et al. [21] to assess the impact of disease and treatment on pediatric patients’ HRQoL during the preceding month. It consists of 23 items divided into four dimensions including physical, emotional, social and school functioning. A 5-point Likert scale was used to estimate problem frequencies: 0 = never, 1 = almost never, 2 = sometimes, 3 = often, 4 = always. Items are then reverse scored and transformed into a scale of 0–100 (0 = 100, 1 = 75, 2 = 50, 3 = 25, 4 = 0), with higher scores representing better HRQoL status. The Total Summary Score was calculated as the sum of all 23 items divided by the number of items answered. The Total Summary Score can be further divided into the subscales of Physical Health Summary Score and Psychosocial Health Summary Score. The Psychosocial Health Summary Score was computed as the sum of 15 items of Emotional, Social and School functioning divided by the number of items answered. This study used self-reports of the Chinese version of the PedsQL™ GCS, which was cross-culturally adapted by Hao et al. [22]. The instrument showed good internal consistency and reliability, with Cronbach’s alpha coefficients of 0.862 in the case group and 0.745 in the control group.

The PedsQL™ FIM was developed by Varin et al. [23] as a parent-reported instrument to measure the impact of pediatric chronic disease on parents’ HRQoL and family functioning. The questionnaire consists of 36 items divided into 8 dimensions including Physical functioning, Emotional functioning, Social functioning, Cognitive functioning, Communication, Worry, Daily activities, and Family relationships. The former 6 dimensions measure parents’ self-reported HRQoL, whereas the latter 2 dimensions measure parent-reported family functioning. Each item has five Likert response options to assess the frequency of problems: 0 (never), 1 (almost never), 2 (sometimes), 3 (often), and 4 (almost). Items are then linearly transformed to a 0–100 scale (0 = 100, 1 = 75, 2 = 50, 3 = 25, 4 = 0), so that higher scores indicate lower family impact. The Total Score is calculated as the sum of all 36 items divided by the number of items answered. The Parent HRQoL Summary Score is calculated as the sum of the 20 items of Physical, Emotional, Social, Cognitive functioning subscales divided by the number of items answered, and the Family Functioning Summary Score is calculated as the sum of the 8 items of daily activities and family relationships subscales divided by the number of items answered. We used the Chinese version of PedsQL™ FIM translated by Chen et al. [24]. It has shown good internal consistency and reliability, with Cronbach’ s alpha coefficients of 0.969 in the case group and 0.943 in the control group.

### Statistical analyses

All data were analyzed using IBM SPSS version 25.0. Continuous and categorical variables were described as mean ± standard deviation (SD) and frequency (percentage n [%]), respectively. First, *t* test and Chi-square (*χ*^2^) test analyses were used to compare the demographic characteristics of the two groups. Second, the *t* test was used to examine differences of the PedsQL™ GCS and PedsQL™ FIM scores between the two groups. And effect size (ES), calculated by Cohen’s *d*, was done to show the differences between two groups on PedsQL™ FIM and PedsQL™ GCS. The ES value were interpreted as: small (0.10–0.30), medium (0.30–0.50), and large (> 0.50) [25]. Third, ANOVA test was used to explore the impact of demographic characteristics on HRQoL of ICPP patients, the variables that were significant at a *P *value of < 0.1 wound be entered to a multiple stepwise regression analysis. Finally, the association between family impact (Total Impact, Parent HQRoL Summary, and Family Functioning Summary) and HRQoL in case group measured by multiple stepwise regression analysis. *P* value < 0.05 (two-sided) was considered statistically significant.

## Results

The demographic characteristics of the case and control groups are summarized in Table 1. A total of 134 were in study group and 210 were controls. The mean age of the study group was 9.05 ± 1.07 years compared to 8.84 ± 1.02 years in controls. One hundred and thirty patients (97.0%) were females which consistent with epidemiologic characteristics of ICPP, whereas 195 controls (92.9%) were females. The mean ages and sex distributions did not differ significantly between the two groups (Table 1). However, height, weight, number of children, parents’ educational level, employment status, and family monthly income were significantly different between the two groups (*P* < 0.05) (Table 1).

**Table 1:** Demographic characteristics of 134 ICPP patients (case group) and 210 controls

Variables	Case group (*N*, %)	Control group (*N*, %)	*t*/*F* value	*P* value
*Patient characteristics*				
Female sex	130 (97.0)	195 (2.9)	2.710	0.100
Age (years)	9.05 ± 1.07	8.84 ± 1.02	1.783	0.076
Height (cm)	139.84 ± 8.36	133.82 ± 8.13	6.624	< 0.001
Weight (kg)	34.73 ± 8.60	30.37 ± 7.13	5.093	< 0.001
Children per family			40.970	< 0.001
One-child	80 (59.7)	53 (25.2)		
Multiple-child	54 (40.3)	157 (74.8)		
Caregivers			3.731	0.053
Parents	125 (93.3)	182 (86.7)		
Grandparents	9 (6.7)	28 (13.3)		
*Caregiver characteristics*				
Living environment			0.037	0.847
Urban	127 (94.8)	200 (95.2)		
Rural	7 (5.2)	10 (4.8)		
Educational level			43.682	< 0.001
Primary school	8 (6.0)	34 (16.2)		
Middle school	11 (8.2)	54 (25.7)		
High school	56 (41.8)	87 (41.4)		
University or higher	59 (44.0)	35 (16.7)		
Employment status			6.833	0.009
Employed	86 (64.2)	162 (77.1)		
Unemployed/housewives	48 (35.8)	48 (22.9)		
Monthly income (CNY)			62.269	< 0.001
< 5000	18 (13.4)	83 (39.5)		
5000–10,000	46 (34.3)	97 (46.2)		
> 10 000	70 (52.2)	30 (14.3)		

The *t* test was used to compare age, height and weight. The Chi-square (*χ*^2^) test was used to compare other variables

*CNY* Chinese Yuan

Table 2 summarizes the clinical characteristics of the case group. Most (91.8%) were at follow-up period, half (66.4%) were diagnosed at age 8–12 years old, and a majority (64.9%) reported a disease duration of less than 1 year. A majority (73.1%) reported a medical treatment duration of less than one year, and patients only using the drug of GnRHa have accounted to 79.9%, most (67.2%) had received intramuscular injection.

**Table 2:** Clinic characteristics of case group (*N* = 134)

Variables	*N* (%)
New diagnosis/follow-up	
New diagnosis	11 (8.2)
Follow-up	123 (91.8)
Age of diagnosis (years)	
5–7	45 (33.6)
8–12	89 (66.4)
Disease duration (years)	
< 1	87 (64.9)
1–2	34 (25.4)
> 2	13 (9.7)
Type of medical treatment	
GnRHa	107 (79.9)
GnRHa + GH	27 (20.1)
Route of administration	
Intramuscular injection	90 (67.2)
Subcutaneous injection	44 (32.8)
Duration of medical treatment (years)	
< 1	98 (73.1)
1–2	29 (21.6)
> 2	7 (5.2)

*GnRHa* gonadotropin release hormone agonist, *GH* growth hormone

Table 3 summarizes the PedsQL^TM^4.0 GCS scores of the two groups. Children with ICPP scored significantly lower than controls in all HRQoL domains except physical functioning. In particular, the two groups were significantly different in emotional functioning scores (77.39 ± 17.97 vs 84.12 ± 14.35, *P* < 0.001).

**Table 3:** Comparison of PedsQL™ 4.0 GCS scores between two groups

Scale	Case group (*N* = 134)	Control group (*N* = 210)	ES (*d*)	95% CI	*t *value	*P *value
	Mean (SD)	Mean (SD)				
Physical health summary score	85.56 ± 11.81	85.54 ± 9.02	0.002	− 0.210 to 0.214	0.017	0.986
Psychosocial health summary scores	82.53 ± 12.17	87.31 ± 9.23	0.443	0.228–0.658	− 3.892	< 0.001
Emotional functioning	77.39 ± 17.97	84.12 ± 14.35	0.414	0.200–0.628	− 3.659	< 0.001
Social functioning	89.10 ± 12.35	92.12 ± 9.28	0.276	0.063–0.489	− 2.421	0.016
School functioning	81.10 ± 14.43	85.69 ± 12.72	0.337	0.124–0.550	− 3.100	0.002
Total summary score	83.28 ± 11.22	86.87 ± 7.59	0.375	0.161–0.589	− 3.261	0.001

Psychosocial health summary scores integrate emotional, social and school functioning scores

Table 4 summarizes the PedsQL™ FIM scores of the two groups. Caregivers of ICPP patients had lower HRQoL and family functioning scores than those caring for healthy children.

**Table 4:** Comparison of PedsQL™ FIM scores between between two groups

	Case group (*N* = 134)	Control group (*N* = 210)	ES (*d*)	95% CI	*t *value	*P *value
	Mean (SD)	Mean (SD)				
Physical functioning	73.38 ± 16.41	81.79 ± 15.34	0.529	0.313–0.745	− 4.822	< 0.001
Emotional functioning	69.74 ± 18.02	79.98 ± 16.92	0.586	0.370–0.802	− 5.338	< 0.001
Social functioning	74.58 ± 17.26	83.48 ± 22.83	0.440	0.225–0.655	− 3.862	< 0.001
Cognitive functioning	72.50 ± 18.08	77.80 ± 18.73	0.288	0.075–0.501	− 2.592	0.010
Communication	77.55 ± 16.48	86.53 ± 16.01	0.553	0.337–0.769	− 5.012	< 0.001
Worry	58.61 ± 18.15	78.03 ± 19.52	1.030	0.804–1.256	− 9.395	< 0.001
Parent HRQOL summary score	72.55 ± 15.64	80.76 ± 15.17	0.532	0.316–0.748	− 4.836	< 0.001
Daily activities	66.98 ± 17.71	73.31 ± 20.54	0.330	0.117–0.543	− 3.034	0.003
Family relationships	74.25 ± 17.40	79.69 ± 18.23	0.305	0.092–0.518	− 2.745	0.006
Family functioning summary score	70.62 ± 15.95	76.50 ± 17.35	0.353	0.140–0.567	− 3.162	0.002
Total score	70.95 ± 14.41	80.07 ± 13.98	0.642	0.425–0.859	− 5.833	< 0.001

Table 5 summarizes that age of patients, BMI, educational level of caregivers, employment status of caregivers, age of diagnosis, disease duration, type of medical treatment, duration of medical treatment significantly influenced HRQoL of ICPP patients in the ANOVA test (all *P* < 0.1).

**Table 5:** ANOVA test between demographic variables and PedsQL™ 4.0 GCS scores in case group

Variables	*N* = 134	Physical functioning	Emotional functioning	Social functioning	School functioning	Psychosocial health	Total
*Patient characteristics*							
Gender							
Male	4	89.06 ± 17.95	67.50 ± 34.03	87.50 ± 13.23	83.75 ± 14.36	79.58 ± 15.72	78.43 ± 14.11
Female	130	86.14 ± 13.27	77.69 ± 17.40	89.15 ± 12.38	81.02 ± 14.48	82.62 ± 12.11	83.43 ± 11.15
Age (years)							
5–7	15	84.17 ± 16.43	72.00 ± 22.10	80.00 ± 14.14^*^	70.47 ± 18.22^*^	74.16 ± 13.74^*^	75.46 ± 12.15^*^
8–12	119	86.49 ± 12.98	78.06 ± 17.37	90.25 ± 11.68	82.44 ± 13.39	83.59 ± 11.59	84.26 ± 10.75
Height^a^							
Normal	116	86.48 ± 13.07	78.02 ± 17.47	89.44 ± 12.54	81.74 ± 14.17	83.07 ± 12.26	83.89 ± 11.24
Abnormal	18	84.62 ± 15.37	73.33 ± 21.00	86.94 ± 11.14	76.94 ± 15.82	79.07 ± 11.22	79.31 ± 10.51
BMI^b^							
Normal	86	86.63 ± 13.31	78.37 ± 17.73	89.01 ± 12.34	81.01 ± 14.76	82.80 ± 12.48^*^	83.36 ± 11.60^*^
Overweight	35	87.27 ± 12.41	78.14 ± 16.89	91.57 ± 11.62	83.43 ± 11.62	84.38 ± 10.23	85.34 ± 9.77
Obesity	13	80.77 ± 15.77	68.85 ± 21.33	83.08 ± 13.16	75.38 ± 18.20	75.77 ± 13.45	77.20 ± 10.94
Children per family							
One-child	80	86.91 ± 12.96	77.94 ± 19.51	90.13 ± 11.33	81.94 ± 14.87	83.33 ± 12.82	84.06 ± 11.76
Multiple-child	54	85.21 ± 13.98	76.57 ± 15.54	87.59 ± 13.69	79.85 ± 13.79	81.34 ± 11.14	82.11 ± 10.37
Caregivers							
Parents	125	86.21 ± 13.25	77.28 ± 18.01	89.40 ± 12.08	81.34 ± 14.39	82.67 ± 12.13	83.42 ± 11.12
Grandparents	9	86.46 ± 15.63	78.33 ± 18.33	85.00 ± 16.01	77.78 ± 15.43	80.56 ± 13.28	81.25 ± 13.06
*Caregiver characteristics*							
Living environment							
Urban	127	86.30 ± 13.38	77.80 ± 17.94	89.09 ± 12.33	81.18 ± 14.26	82.69 ± 10.09	83.43 ± 11.21
Rural	7	84.82 ± 13.91	70.00 ± 18.26	89.29 ± 13.67	79.57 ± 18.52	79.62 ± 14.15	80.58 ± 11.90
Educational level							
Primary school	8	80.08 ± 13.56	70.63 ± 17.20	81.88 ± 7.04^*^	80.63 ± 11.16^*^	77.71 ± 8.50^*^	78.01 ± 7.02^*^
Middle school	11	86.93 ± 8.48	68.18 ± 24.21	84.09 ± 13.38	70.90 ± 17.58	74.39 ± 14.65	75.40 ± 12.18
High school	56	87.22 ± 12.84	79.02 ± 19.64	91.52 ± 10.83	81.07 ± 14.82	83.87 ± 12.62	84.30 ± 11.68
University or higher	59	85.99 ± 14.55	78.47 ± 14.54	88.73 ± 13.57	83.08 ± 13.31	83.43 ± 11.15	84.49 ± 10.48
Employment status							
Employed	85	87.22 ± 12.97	80.65 ± 15.19^*^	89.71 ± 12.47	82.73 ± 13.39^*^	84.36 ± 11.09^*^	84.99 ± 10.41^*^
Unemployed/housewives	49	84.50 ± 13.96	71.73 ± 20.96	88.06 ± 12.20	78.27 ± 15.83	79.35 ± 13.36	80.31 ± 12.05
Monthly income (CNY)							
≤ 5000	18	86.11 ± 13.27	76.39 ± 16.16	91.67 ± 10.00	81.11 ± 15.58	83.06 ± 11.49	83.17 ± 10.94
5000–10,000	46	84.47 ± 14.95	76.09 ± 19.66	86.85 ± 13.80	78.63 ± 15.83	80.52 ± 13.67	81.26 ± 12.78
≥ 10,000	70	87.41 ± 12.29	78.50 ± 17.41	89.93 ± 11.81	82.71 ± 13.10	83.71 ± 11.25	84.63 ± 10.10
*Clinic characteristics of patients*							
New diagnosis/follow-							
New diagnosis	11	92.33 ± 9.10	84.09 ± 12.41	90.00 ± 8.06	79.55 ± 11.93	84.55 ± 6.54	84.64 ± 5.89
Follow-up	123	85.68 ± 13.56	76.79 ± 18.30	89.02 ± 12.69	81.24 ± 14.67	82.35 ± 12.55	83.15 ± 11.59
Age of diagnosis (years)							
5–7	45	85.42 ± 13.31	75.44 ± 16.27	85.11 ± 13.16^*^	79.27 ± 13.85	79.94 ± 11.06^*^	80.63 ± 9.94^*^
8–12	89	86.64 ± 13.43	78.37 ± 18.78	91.12 ± 11.48	82.02 ± 14.71	83.84 ± 12.55	84.61 ± 11.64
Disease duration (years)							
≤ 1	87	88.30 ± 12.54^*^	77.59 ± 19.39	89.71 ± 12.16	81.15 ± 15.03	82.82 ± 12.91	83.76 ± 11.77
1–2	34	80.51 ± 14.00	76.18 ± 14.88	87.94 ± 12.38	79.32 ± 14.14	81.15 ± 10.51	81.63 ± 9.84
≥ 2	13	87.26 ± 13.65	79.39 ± 16.31	88.08 ± 14.22	85.38 ± 10.50	84.23 ± 11.54	84.33 ± 11.21
Type of medical treatment							
GnRHa	107	85.99 ± 13.89	77.01 ± 12.28	87.20 ± 12.83^*^	80.25 ± 14.67	81.49 ± 12.12^*^	82.13 ± 11.19^*^
GnRHa + growth hormone	27	87.15 ± 11.18	78.89 ± 20.78	96.67 ± 5.88	84.44 ± 13.18	86.67 ± 11.66	87.83 ± 10.30
Route of administration							
Intramuscular injection	90	85.52 ± 13.69	78.17 ± 18.26	89.28 ± 12.42	81.44 ± 13.68	82.96 ± 11.63	83.45 ± 10.65
Subcutaneous injection	44	87.67 ± 12.70	75.80 ± 17.45	88.75 ± 12.35	80.39 ± 16.00	81.64 ± 13.29	82.91 ± 12.42
Duration of medical treatment (years)							
≤ 1	98	87.96 ± 12.11^*^	76.99 ± 18.79	89.39 ± 12.17	81.28 ± 14.75	82.55 ± 12.62	83.47 ± 11.54
1–2	29	80.39 ± 15.19	77.41 ± 15.96	88.97 ± 11.83	79.38 ± 13.66	81.92 ± 10.49	82.18 ± 9.84
≥ 2	7	86.16 ± 17.01	82.86 ± 14.96	85.71 ± 17.90	85.71 ± 13.67	84.76 ± 13.59	85.11 ± 13.29

^a^Height of patient is higher than the same age and sex 97th percentile (> P97) is recorded as abnormal value, in normal range or below normal range is recorded as normal value according to the standardized curve of height and body mass of children and adolescents aged 0–18 years in China

^b^The Body Mass Index (BMI) is divided into normal group, overweight group and obesity group according to the growth curve of body mass index of children and adolescents aged 0–18 years in China

^*^P < 0.1

Variables that were significant at a *P* value of < 0.1 in the ANOVA test were selected for the final multiple stepwise regression model. Table 6 summarizes that parent HRQoL was positively associated with the domains of physical functioning, school functioning, psychosocial functioning, and total HRQoL in ICPP patients (beta: 0.181–0.242, *P* < 0.05). Family functioning was positively associated with the domain of social functioning in ICPP patients (beta: 0.190, *P* = 0.018). Employment status of caregivers was negatively associated with the domains of emotional functioning and psychosocial health in ICPP patients (beta: − 0.177 to − 0.240, *P* < 0.05). Age of patients was positively associated with the domain of social functioning, school functioning, psychosocial functioning and total HRQoL in ICPP patients (beta: 0.185–0.250, *P* = 0.018). Type of medical treatment was positively associated with the domains of social functioning and total HRQoL in ICPP patients (beta: 0.188–0.273, *P* = 0.018). Educational level of caregivers was positively associated with total HRQoL in ICPP patients (beta: 0.167, *P* = 0.042).

**Table 6:** Multiple stepwise regression analysis for family impact, demographic variables and HRQoL in ICPP patients

Variables	Unadjusted		Adjusted	*t *value	*P *value
	*B*	SE	Beta		
Physical health summary score					
Parent HRQOL	0.163	0.064	0.216	2.536	0.012
Emotional functioning					
Employment status	− 8.912	3.141	− 0.240	− 2.838	0.005
Social functioning					
Type of medical treatment	8.364	2.452	0.273	3.411	0.001
Age	8.631	3.119	0.221	2.767	0.006
Family functioning	0.147	0.062	0.190	2.395	0.018
School functioning					
Age	11.408	3.759	0.250	3.035	0.003
Parent HRQOL	0.194	0.076	0.210	2.545	0.012
Psychosocial health					
Age	9.183	3.145	0.239	2.920	0.004
Employment status	− 4.445	2.080	− 0.177	− 2.137	0.031
Parent HRQOL	0.141	0.064	0.181	2.187	0.034
Total					
Age	6.573	2.899	0.185	2.267	0.025
Type of medical treatment	5.236	2.249	0.188	2.328	0.021
Educational level	2.218	1.081	0.167	2.053	0.042
Parent HRQOL	0.174	0.058	0.242	3.012	0.003

## Discussion

This case–control study in Chongqing, China revealed that children with ICPP demonstrated lower quality of life, and disease and its treatment have possibly negative effects on parental HRQoL and family functioning.

Our study revealed that children with ICPP performed worse than healthy peers in emotional functioning, social functioning, school functioning, psychosocial health, and total HRQoL using the PedsQL^TM^4.0 GCS instrument. Similarly, a study using an *Inventory of Subjective Life Quality Scale* found that ICPP patients had worse scores on domains of school life, depression, anxiety, and body satisfaction than healthy controls [15]. However, a study in Australia showed that children who experienced early puberty scored poorly only on PedsQL™ psychosocial health summary scores, but had similar scores in other dimensions as controls [16]. These discrepant results may be related to the different cultural adaptations of the instruments. The highly significant difference in the emotional functioning of the two groups can be attributed to physical changes caused by ICPP. Children with ICPP were burdened by physical changes such as abnormally tall stature, and precocious breast development and menarche in girls, these changes may lead to mental and behavioral problems, which include depression, low self-concept, withdrawal, somatization, social problems, and delinquent and aggressive behaviors [26, 27]. These psychological and behavioral problems of children with ICPP may impair their social development. A qualitative research study found that children with ICPP experienced feelings of isolation and being bullied, and displayed aggressive bullying behavior [14]. Although physical differences that are caused by secondary sexual characteristics may be temporary, the impact on the child’s feelings of alienation from the peer group may be significant [28]. In addition, children with ICPP require therapeutic injections at 4 to 5-week intervals and examinations every 3 months that may subsequently impede school attendance. However, the physical functioning scores were similar in the two groups in our study. This finding may be related to physical changes in children with ICPP that may be advantageous in physical activities such as sports, such as taller height caused by bone maturation.

The impact of chronic diseases of children such as cancer [29], sickle cell disease [30], chronic pain [31], and functional constipation [32] on parent HRQoL and family functioning has been demonstrated repeatedly. This study also demonstrated that caring for a child with ICPP is associated with lower parental HRQoL and impaired family functioning in all dimensions of the FIM. Primary caregivers must balance the management of illness with housework, they may sacrifice their social life for better management of illness. Daily administration of growth hormone injections, as well as supervision of the activities of daily life that include diet, sports, and sleep made them physical discomfort [33]. Caregivers also expressed negative emotions and reported stressors such as medication side effects, diagnostic uncertainly, and financial costs that increased their psychological burden [12].

Our study also found that parents of patients had lower family functioning scores than controls. In China, older relatives including fathers may misperceive ICPP as a normal developmental process, and conclude that medical treatment is unnecessary, while mothers may advocate timely treatment for their children; consequently, family members may disagree on the child’s treatment. Another probable explanation for lower family functioning may be that family members must sacrifice family activities to take care of their child with ICPP. Under the view of family-centered pediatric nursing, health care professionals can not ignore the negative impact of ICPP on parent HRQoL and family functioning.

There were multiple factors associated with HRQoL of ICPP patients. Age of patients, type of medical treatment, employment status and educational level of caregivers, parent HRQoL and family functioning were all significantly associated with some of domains of HRQoL in case group.

In our study, older patients reported better HRQoL. Older patients can better deal with the impact of illness on their daily life with increased cognitive ability and disease knowledge [34]. Lower educational level and unemployment status of caregivers were also the important risk factors for the Total HRQoL as well as domains of Emotional functioning and Psychosocial Health in ICPP patients. Patients living in this kind of family circumstances may be more likely to express negative emotions [35]. On the other hand, a well-educated caregiver has a strong ability to receive disease-related knowledge, which can provide comprehensive care for the patients [36]. Type of treatment is another factor associated with HRQoL of ICPP patients. Our study found that ICPP patients treated with drug combination reported better HRQoL in the Social functioning. One may hypothesize that the growth rate was obviously improved in the group of drug combination [37, 38], making them more confident in their social life.

In our study, less impact of disease on parent HRQoL was associated with better HRQoL in the Physical functioning, School functioning and Psychosocial Health domains in ICPP patients. On the one hand, children may learn the coping strategies of disease and treatment through their parents, and caregivers with good physical and mental health are likely to adopt a positive approach to the disease management [39], so their children have higher treatment compliance and less impact on physical and psychsocial health. On the other hand, caregivers who were in good physical and mental health may have more time and energy to deal with their children’s school life. In addition, the less impact of disease on family functioning, the better HRQoL in the social functioning of patients. Healthy family functioning such as harmonious family relationships and ability of solving problems promoted good social adaptation of patients [40].

However, this study had several limitations. First, the PedsQL^TM^4.0 GCS is a general questionnaire and may lack of precision and sensitivity in assessing HRQoL of children with ICPP. However, this Chinese version of PedsQL^TM^4.0 GCS has acceptable psychometric properties, and many researchers have used it to evaluate patients with other endocrinopathies such as obesity and short stature. Second, because our case group was recruited primarily from a triple A children’s hospital in Chongqing, their HRQoL may not be representative of those of children with ICPP from other regions. However, our study found that ICPP impacts the HRQoL of both patients and their parents, as well as family functioning. Similar studies are needed in other regions of China. Finally, it may be difficult to compare the HRQoL of paient aged 5 and 12 because of the large age range of sample.

## Conclusion

We conclude that ICPP patients demonstrated lower quality of life, and disease and its treatment have possibly negative effects on parental HRQoL and family functioning. In addition, the age of patients, type of medical treatment, employment status and educational level of caregivers, parent HRQoL and family functioning were all significantly associated with some of domains of HRQoL in case group. These findings suggest that health care professionals should pay more attention to younger patients treated with GnRHa alone, and provide targeted interventions for caregivers depending on their demographic background to reduce family impact and thereby improve patient HRQoL.

## Data Availability

The data used or analyzed during the current study are available from the corresponding author on reasonable request.

## Abbreviations

ICPP: Idiopathic central precocious puberty
HRQoL: Health-related quality of life
PedsQL™ GCS: PedsQL™ Generic Core Scales
FIM: Family impact module
GnRHa: Gonadotropin releasing hormone agonists
CNY: Chinese Yuan
SD: Standard deviation
GH: Growth hormone

## References

[1] Latronico AC, Brito VN, Carel JC (2016). Causes, diagnosis, and treatment of central precocious puberty. Lancet Diabetes Endocrinol.

[2] Elizabeth RG (1982). For puberty that comes too soon, new treatment highly effective. JAMA.

[3] Teilmann G, Pedersen CB, Jensen TK (2005). Prevalence and incidence of precocious pubertal development in Denmark: an epidemiologic study based on national registries. Pediatrics.

[4] Zhu MQ, Fu JF, Liang L (2013). Epidemiologic study on current pubertal development in Chinese school-aged children. Zhejiang Univ (Med Sci).

[5] Carel JC, Eugster EA, Rogol A (2009). Consensus statement on the use of gonadotropin-releasing hormone analogs in children. Pediatrics.

[6] Swaiss HH, Khawaja NM, Farahid OH (2017). Effect of gonadotropin-releasing hormone analogue on final adult height among Jordanian children with precocious puberty. Saudi Med J.

[7] Ying Y, Tang J, Chen W (2017). GnRH agonist treatment for idiopathic central precocious puberty can improve final adult height in Chinese girls. Oncotarget.

[8] Choi MS, Kim EY (2016). Body image and depression in girls with idiopathic precocious puberty treated with gonadotropin-releasing hormone analogue. Ann Pediatr Endocrinol Metab.

[9] Wojniusz S, Callens N, Sütterlin S (2016). Cognitive, emotional, and psychosocial functioning of girls treated with pharmacological puberty blockage for idiopathic central precocious puberty. Front Psychol.

[10] Baumann DA, Landolt MA, Wetterwald R (2001). Psychological evaluation of young women after medical treatment for central precocious puberty. Horm Res.

[11] Zheng F, Zhu H, Jiang YJ (2008). Psychological behavior of girls with idiopathic central precocious puberty before and after treatment with gonadotropin-releasing hormone analogue. Zhejiang Univ (Med Sci).

[12] Zhu S, Yu YZ, Shu S (2014). Qualitative research on load of mother care for children with idiopathic central precocious puberty. Chin Nurs Res.

[13] Xie CH, Tang JY (2017). Investigation of parental stress, social support and quality of life in mothers of children with precocious puberty. Chin J Mod Nurs.

[14] Patel PS (2016). Identifying areas of needed support for young girls with central precocious puberty (CPP).

[15] Liu YY, Ma YP, Jin ZY (2015). A relevant research on quality of life and self-awareness in girls with idiopathic central precocious puberty. Chin J Behav Med Brain Sci.

[16] Mensah FK, Bayer JK, Wake M (2013). Early puberty and childhood social and behavioral adjustment. J Adolesc Health.

[17] Hu LP (2010). SAS experimental design and statistical analysis.

[18] Yuan JN, Liang L, Cai XD (2011). Impact of gonadotropin-releasing hormone analogs on body mass index in girls with idiopathic central precocious puberty: a long-term follow-up study. Chin J Contemp Pediatr.

[19] Zhu DQ, Zhu H (2016). The status survey of children with precocious puberty in Chinese cities. Chin Pediatr Integr Tradit West Med.

[20] Liang L, Du ML, Luo XP (2015). Consensus on diagnosis and treatment of central precocious puberty. Chin J Pediatr.

[21] Varni JW, Thompson KL, Hanson V (1987). The Varni/Thompson pediatric pain questionnaire: I. Chronic musculoskeletal pain in juvenile rheumatoid arthritis. Pain.

[22] Lu YY, Tian Q, Hao YT (2008). Reliability and validity for Chinese version of pediatric quality of life inventory PedsQL4.0. SUN-Yat-sen Univ (Med Sci)..

[23] Varni JW, Sherman SA, Burwinkle TM (2004). The PedsQL^TM^ Family Impact Module: preliminary reliability and validity. Health Qual Life Outcomes.

[24] Chen R, Hao Y, Feng L (2011). The Chinese version of the Pediatric Quality of Life Inventory^TM^ (PedsQL^TM^) family impact module: cross-cultural adaptation and psychometric evaluation. Health Qual Life Outcomes.

[25] Cohen J (2013). Statistical power analysis for the behavioral sciences.

[26] Qiao XH, Yu J, Xie XT (2008). Case-control Study on behavioral problems of girls with earlier onset of sexual development. Chin Ment Health J.

[27] Ai LL, Jia SM, Zhang YX (2018). Investigation on the self-concept of children with precocious puberty. Chin J Child Health Care.

[28] Golum MS, Collman GW, Foster PM (2008). Public health implications of altered puberty timing. Pediatrics.

[29] Scarpelli AC, Paiva SM, Pordeus IA (2008). The pediatric quality of life inventory (PedsQL) family impact module: reliability and validity of the Brazilian version. Health Qual Life Outcomes.

[30] Panepinto JA, Hoffmann RG, Pajewski NM (2009). A psychometric evaluation of the PedsQL Family Impact Module in parents of children with sickle cell disease. Health Qual Life Outcomes.

[31] Mano KEJ, Khan KA, Ladwig RJ (2011). The impact of pediatric chronic pain on parents' health-related quality of life and family functioning: reliability and validity of the PedsQL 4.0 Family Impact Module. J Pediatr Psychol.

[32] Wang CJ, Shang L, Zhang YH (2013). Impact of functional constipation on health-related quality of life in preschool children and their families in Xi’an, China. PLoS ONE.

[33] Zhang XX, Yi ZW, Zhang JJ (2005). A preliminary study on mental health in children with precocious puberty and their parents. Chin J Clin Psychol.

[34] Ou XW (2003). Psychological characteristics of children with chronic diseases.

[35] Spencer N (2006). Socioeconomic determinants of health related quality of life in childhood and adolescence: results from a European study. Child Care Health Dev.

[36] Zhang HL (2015). The quality of life and its influencing factors among adolescents with systematic lupus erythematosus.

[37] Jung MK, Song KC, Kwon AR (2014). Adult height in girls with central precocious puberty treated with gonadotropin-releasing hormone agonist with or without growth hormone. Ann Pediatr Endocrinol Metab.

[38] Lewis KA, Goldyn AK, West KW (2013). A single histrelin implant is effective for 2 years for treatment of central precocious puberty. J Pediatr.

[39] Rhee SJ, Kim EY, Kim SH (2014). Subjective depressive symptoms and metabolic syndrome among the general population. Prog Neuropsychopharmacol Biol Psychiatry.

[40] Zhang DJ, Zhu ZG, Liu GZ (2019). The relationship between social support and problem behaviors of adolescents: the chain mediating role of psychological suzhi and self-esteem. J Southwest Univ (Soc Sci Ed).

